# Transarterial infusion chemotherapy for advanced esophageal cancer with airway stenosis

**DOI:** 10.3389/fonc.2023.1238287

**Published:** 2023-08-30

**Authors:** Gang Zhou, Meipan Yin, Wei He, Yaozhen Ma, Chunxia Li, Zhen Li, Xiaobing Li, Shuai Wang, Gang Wu

**Affiliations:** ^1^ Department of Interventional Radiology, The First Affiliated Hospital of Zhengzhou University, Zhengzhou, China; ^2^ Oncology Department, The First Affiliated Hospital of Zhengzhou University, Zhengzhou, China

**Keywords:** esophageal cancer, airway stenosis, transarterial infusion chemotherapy, dyspnea, interventional radiology

## Abstract

**Purpose:**

This study aimed to investigate the safety and efficacy of transarterial infusion chemotherapy for the treatment of esophageal cancer with airway stenosis.

**Methods:**

Data of patients with advanced esophageal cancer complicated with airway stenosis treated with transarterial infusion chemotherapy were retrospectively analyzed. Dyspnea, clinical efficacy and adverse reactions were evaluated.

**Results:**

Of these patients, 27 had grade II preoperative dyspnea, and 31 had grade III preoperative dyspnea, 26 had grade I postoperative dyspnea, 25 had grade II postoperative dyspnea, and 7 had grade III postoperative dyspnea. Among 3 patients with left main bronchial stenosis and atelectasis, 2 had complete remission after transarterial infusion chemotherapy, and 1 demonstrated partial remission. After treatment, complete response, partial response, and stable disease were observed in 7, 34, and 17 cases, respectively. Total objective effective rate and disease control rate were 70.6% (41/58) and 100.0%, respectively. During follow up, 24 patients died of organ failure, and 17 patients died of tumor-related respiratory failure. Seven patients died of gastrointestinal bleeding, 1 patient died of myocardial infarction, and 9 patients survived.

**Conclusions:**

Transarterial infusion chemotherapy is safe and effective for the treatment of advanced esophageal cancer with airway stenosis.

## Introduction

The clinical treatment of esophageal cancer with airway stenosis is difficult, since radical resection of esophageal cancer with airway stenosis cannot be performed (1)**. **Intravenous chemotherapy is the first choice of treatment for unresectable esophageal cancer, but the effective rate is low (8%–54%) (2, 3). Advanced esophageal cancer is not sensitive to radiotherapy, and radiotherapy can cause acute radiation pneumonitis, which aggravates airway stenosis (4, 5).

Airway stents can rapidly expand the narrow lumen and relieve dyspnea; thus, they have been widely used in the clinical treatment of benign and malignant airway stenosis (6, 7). Transarterial infusion chemotherapy (TAIC) can be used to directly inject chemotherapeutic drugs into the tumor area through an artery, which can greatly increase the drug concentration in the tumor and improve the curative effect, but it is rarely used for the treatment of esophageal cancer (8–10). The purpose of this study was to investigate the safety and efficacy of TAIC for the treatment of esophageal cancer with airway stenosis.

## Material and methods

The clinical data for all consecutive patients with esophageal cancer complicated with airway stenosis treated at our interventional treatment center from November 2014 to January 2023 were retrospectively analyzed, including medical records, imaging, interventional surgery, and follow-up data. The inclusion criteria were as follows: 1) pathological diagnosis of esophageal cancer and imaging confirmation of esophageal cancer with airway stenosis; 2) esophageal carcinoma and airway stenosis treated with TAIC. The exclusion criteria were as follows: 1) airway stenosis caused by non-esophageal cancer; 2) absence of TAIC; 3) airway stenosis caused by esophageal cancer treated with airway stents. The institutional ethics committee approved this research, which complies with the principles set out in the Declaration of Helsinki. Written informed consent was obtained from all patients. Ethical approval code: SS-2018-22.

### TAIC

#### Preoperative preparation

**Figure 1:**
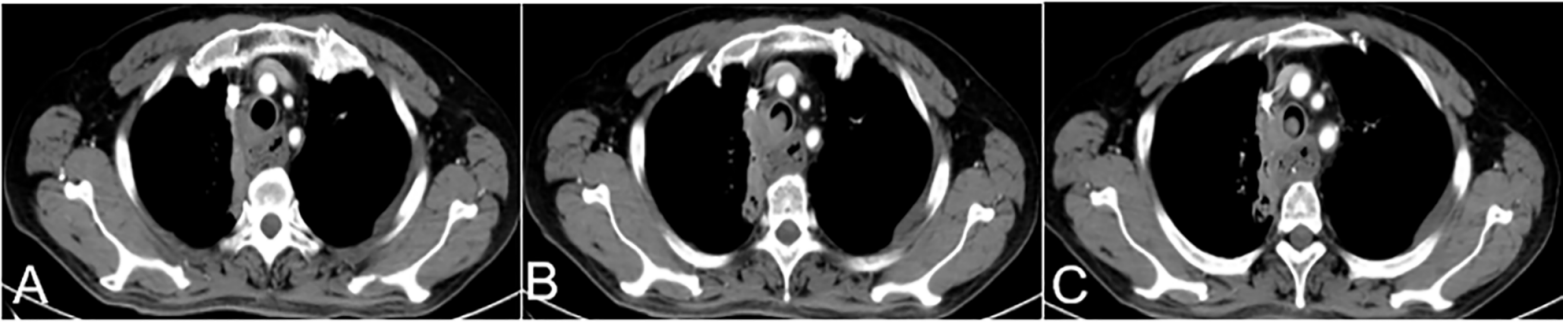
A 63-year-old female with hemoptysis and dyspnea more than half a month after esophageal cancer surgery. **(A–C)** Preoperative CT showed a soft tissue density shadow on the right side of the trachea at the thoracic entrance, protruding into the trachea. This soft tissue density shadow corresponded to stenosis of the middle trachea, which was evenly enhanced CT, computed tomography.

Preoperative blood tests, liver and kidney function tests, electrolyte measurements, electrocardiography, and chest enhanced computed tomography (CT) were performed to evaluate the physical strength and nutritional status of patients (**Figure 1**).

The degree of dyspnea was evaluated before TAIC. Patients could lie flat under oxygen inhalation, and dyspnea could be tolerated.

### Procedure

**Figure 2:**
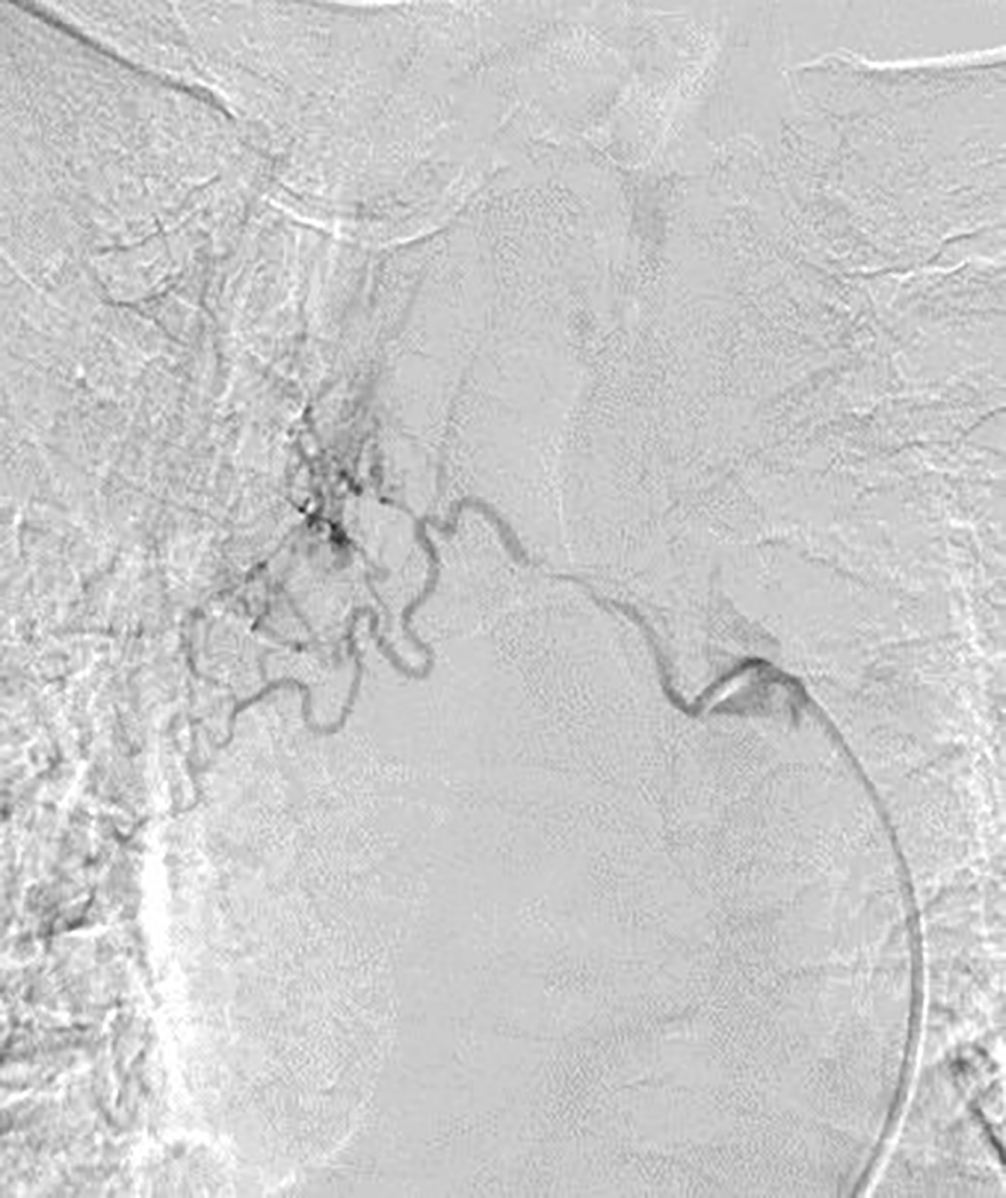
DSA showed that the right bronchial artery was tortuous and thickened, and abnormal vascular branches supplied lung tissue. DSA, digital subtraction angiography.

Patients assumed a supine position on the digital subtraction angiography table. Patients were awake, and local anesthesia was applied at the right femoral artery puncture point. Femoral artery puncture was performed using a 5-F arterial sheath. A 5-F Cobra catheter or vertebral artery catheter was introduced through the sheath to find the supporting artery corresponding to the lesion. According to each patient’s body surface area and physical condition, Adriamycin (30–50 mg), oxaliplatin (100 mg), and raltitrexed (4 mg) were administered, and each chemotherapy drug was prepared in 150–200 ml of diluted solution with the appropriate compatibility solution. According to the blood supply of target vessels, the doses of perfusion chemotherapy drugs were reasonably adjusted, and the perfusion time of each drug was maintained at 15–20 min (**Figure 2**).

### Postoperative management

**Figure 3:**
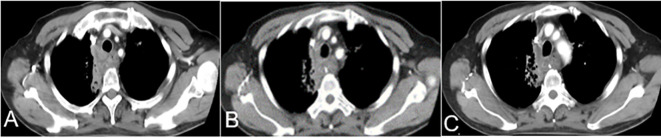
One month after TAIC, **(A–C)** CT showed a soft tissue density shadow on the right side of the trachea at the thoracic entrance, protruding into the trachea. Middle tracheal stenosis had improved. CT, computed tomography; TAIC, transarterial infusion chemotherapy.

Patients were treated with antiemetic drugs, acid suppression, and hydration therapy. Bloods, liver and kidney function, electrolytes, and other indicators were monitored 7 days after surgery. If white blood cell and platelet counts were low, white blood cells and platelets were administered. One month after surgery, chest CT was reexamined to evaluate the curative effect (**Figure 3**).

### Evaluation criteria for clinical efficacy and adverse reactions

According to the dyspnea classification standard of the American Thoracic Association (ATA), airway stenosis grading and its changes were evaluated before and 7 days after TAIC.

Clinical staging of all patients before and after treatment was evaluated according to the criteria for clinical staging of the American Joint Committee on Cancer (11). The clinical efficacy of drugs used to treat esophageal cancer was evaluated according to the complete response (CR), partial response (PR), and presence of stable disease (SD) or progressive disease (PD) (12, 13). CR+PR was defined as the objective response rate (ORR). Disease control rate (DCR) was defined as CR+ PR + SD. If the curative effect was a CR, then conversion to radiotherapy was indicated. If the curative effect was a PR or SD, plus perfusion chemotherapy was administered. If the lesion demonstrated PD, other types of palliative treatment were indicated.

Adverse reactions of chemotherapy drugs were recorded. The toxicity and side effects of chemotherapy drugs were evaluated according to National Cancer Institute Common Terminology Criteria for Adverse Events (NCI-CTCAE, version 4.0) and classification of anticancer-drug toxicity (0–IV).

### Statistical analysis

Data were analyzed using Prism 5 (GraphPad, San Diego, CA, USA). Data are presented as mean ± standard deviation. The postoperative dyspnea grading was compared using the rank-sum test. A *P*-value of <0.05 was considered statistically significant.

## Results

Fifty-eight patients with esophageal carcinoma with airway stenosis were included, including 32 males and 26 females with an age range of 46–86 years (mean, 64.6 ± 8.8 years) (**Table 1**).

**Table 1:** Patient demographics.

Sex	n (%)
Male	32 (55.2%)
Female	26 (44.8%)
Age (years)	
≥75	9 (15.6%)
<75	49 (84.4%)
Location of esophageal cancer	
Upper-segment lesions	21 (36.2%)
Middle-segment lesions	16 (27.6%)
Lower-segment lesions	3 (5.2%)
Anastomotic region tumor recurrence	18 (31.0%)
Narrow position	
Main airway	33 (56.9%)
Carina	5 (8.6%)
Left main branch	17 (29.3%)
Right main bronchial	3 (5.2%)
Was there associated lung collapse?	
Yes	4 (6.9%)
No	54 (93.1%)
Complications	
Esophagotracheal fistula	20 (34.5%)
Esophagomediastinal fistula	2(3.4%)
Esophagogastric anastomotic fistula	1(1.7%)
Comorbidities	
Hypertension	11 (19.0%)
Diabetes mellitus	8 (13.8%)

Twenty patients with esophageal cancer and esophageal fistula were treated with conservative therapy (i.e., nutrition tube placement) before TAIC. Three patients with esophageal cancer and esophageal fistula were treated with a covered esophageal stent before TAIC.

In 58 patients, the feeding artery of the tumor was identified and perfused with chemotherapy drugs. For each patient, 1–4 feeding arteries were perfused, including the bilateral inferior thyroid artery (13 cases), the bilateral bronchial artery (30 cases), the unilateral bronchial artery (24 cases), the proper esophageal artery (6 cases), the intercostal artery (20 cases), the right gastroepiploic artery (7 cases), the thyroid artery(4 cases), the right internal thoracic artery (2 cases), and the right gastric artery(3 cases). Intraoperatively, a microcatheter was used in 55 cases for super-selective intubation to protect blood vessels, as well as to avoid injury to spinal arteries and drug reflux. Thirty-six patients received one course of TAIC, 16 patients received two courses of TAIC, and 6 patients received three courses of TAIC.

### Evaluation of clinical efficacy

After TAIC, the tumor focus was confirmed by chest CT, and the degree of airway stenosis was alleviated to varying degrees. Among the 4 patients with left main bronchial stenosis and atelectasis, the lung of 3 cases was demonstrated to be completely open by CT after TAIC, and the lung of 1 case was partially open after TAIC. According to the ATA classification of dyspnea, there were 27 cases of grade II dyspnea and 31 cases of grade III dyspnea preoperatively. One week after surgery, oxygen saturation was >95% without oxygen inhalation, and 26 cases of grade I dyspnea, 25 cases of grade II dyspnea, and 7 cases of grade III dyspnea were observed. Compared with before surgery, the dyspnea grading was lower 1 week after surgery (z = 6.1, *p* < 0.001). One patient was complicated with esophagogastric anastomotic leakage before TAIC. Nutrition and gastrointestinal decompression tubes were inserted, and TAIC was performed once. After TAIC, esophagogastric anastomotic leakage was completely healed 3 weeks later. Four patients were complicated with tracheoesophageal fistula before TAIC. Nutrition and gastrointestinal decompression tubes were inserted, and TAIC was performed twice simultaneously. The fistula healed after 1 month (**Table 2**).

**Table 2:** Pre- and post-TAIC dyspnea grading.

	I	II	III
Pre-TAIC	0	27	31
Post-TAIC	26	25	7

TAIC, transarterial infusion chemotherapy.

The evaluation of target lesions in patients before treatment was T3 (9 cases), and T4 (49 cases). After 1–3 courses of treatment, 58 patients were followed up, and the clinical stages were T1 (14 cases), T2 (13 cases), T3 (14 cases), and T4 (17 cases). After treatment, the evaluation of target lesions in patients decreased significantly (**Table 3**).

**Table 3:** Clinical classification before and after TAIC.

Classification	Before treatment	After the first course	After the second course	After the third course
n	58	58	22	6
T1	0	10	4	0
T2	0	13	6	0
T3	9	16	6	4
T4	49	19	6	2

TAIC, transarterial infusion chemotherapy.

After the first course of TAIC, a CR was noted in 4 cases, a PR was noted in 32 cases, SD was noted in 22 patients, and the ORR was 62.1%. Twenty-two patients received a second course of TAIC for esophageal cancer. After the second course, a CR was noted in 3 cases, a PR was noted in 16 cases, SD was noted in 3 cases, and the ORR was 86.4%. Six patients received a third course of TAIC for esophageal cancer. After the third course, a CR was noted in 0 cases, a PR was noted in 4 cases, SD was noted in 2 cases, and the ORR was 74.0%. After 1–3 courses of treatment, a CR was noted in 7 cases, a PR was noted in 34 patients, SD was noted in 17 cases, the ORR was 70.7%, and the DCR was 100%.

### Complications

Grade I–III adverse reactions occurred after TAIC for esophageal cancer (**Table 4**). These and other common adverse reactions were relieved quickly after symptomatic treatment.

**Table 4:** Adverse reactions after transarterial infusion chemotherapy.

	I	II	III
Reduction in white blood cell count	6	1	2
Thrombocytopenia	2	3	0
Vomiting	27	5	1
Fever	3	2	2

### Follow-up

The median follow-up time was 15.4 months. Twenty-four patients died of systemic organ failure, and 17 patients died of tumor-related respiratory failure. Seven patients died of gastrointestinal bleeding due to tumor rupture. One 86-year-old patient died of myocardial infarction 18 months after TAIC.

At the end of follow-up, 9 patients were alive. All 9 patients are free from dyspnea, and 1 patient with dysphagia took food through a nasal nutrition tube. Among the surviving patients, 2 received postoperative radiotherapy and 3 received postoperative immune-targeted therapy.

## Discussion

Esophageal cancer directly invades the trachea and main bronchus, leading to airway stenosis or lymph node metastasis, enlargement, and airway compression (14–16). If we do not actively and effectively control disease development, dyspnea may become aggravated, infection may occur after obstruction, and asphyxia and death may be observed (17).

Clinically, the treatment options for advanced esophageal cancer with airway stenosis are limited. Palliative treatment, such as endoscopic local treatment, laser therapy, thermal ablation, cryotherapy, airway stent placement, and photodynamic therapy, can be used to treat airway stenosis (1, 16, 18, 19). Intravenous chemotherapy is one of the standard treatment options for patients with advanced esophageal cancer. However, patients with advanced esophageal cancer complicated with airway stenosis are generally in a poor condition, and they cannot tolerate intravenous chemotherapy. In addition, chemotherapy cannot quickly relieve the symptoms of dyspnea (20–22). Airway stenting cannot control the growth of tumor tissue. It also only temporarily relieves airway stenosis and cannot treat primary disease. However, it has been reported that airway stenting combined with other therapies for primary disease can significantly improve survival of patients (23–25). However, airway stent implantation has complications, including airway restenosis, airway bleeding, stent displacement, and stent rupture, amongst others (26). Therefore, for patients with esophageal cancer with mild or moderate dyspnea who can tolerate direct TAIC, we should first treat primary disease to reduce the focus quickly and alleviate dyspnea, which avoids the complications associated with airway stent implantation.

Compared with systemic intravenous chemotherapy, TAIC for esophageal cancer uses a higher concentration of chemotherapy drugs that directly act via the tumor blood supply artery, which can rapidly reduce tumor size and alleviate airway stenosis without the need for airway stent implantation. This approach avoids a series of complications caused by airway stenting and has the advantage of reducing toxicity and side effects. Yin et al. (8) reported that 75 patients underwent 1–3 cycles of TAIC, and the total effective rate (CR + PR) was 94.7%, 13 patients had airway stenosis before TAIC, and no airway stents were inserted. After TAIC, tumor size was significantly reduced, and the symptoms of dyspnea were significantly alleviated. After TAIC, the degree of airway stenosis in the 50 patients studied at our center was alleviated to varying degrees. The dyspnea classification standard was lower 1 week after TAIC compared with before TAIC, and the clinical stage of tumors was significantly lower.

According to the location of esophageal cancer lesions, identifying all tumor-feeding arteries is the key to TAIC. Feeding arteries in esophageal cancer are changeable and complex, but there are certain rules to follow. According to the location of esophageal cancer, feeding arteries can determined (8). Choosing the right type of catheter, carefully identifying the tumor-feeding artery, and using a microcatheter to super-selectively intubate when necessary, can effectively improve the concentration of anticancer drugs in the tumor area, reduce the damage of chemotherapy drugs to non-target vessels, and effectively prevent misperfusion. Regular treatment of tumor cells in each cycle can increase the effect of chemotherapeutic drugs, destroy the formation and growth of tumor blood vessels, and consolidate the curative effect. For patients who cannot feed normally by mouth, timely placement of a jejunal nutrition tube can ensure nutritional support, which is conducive to patient recovery.

One limitation of this study is that it was performed at a single center. While the sample size was not small, it may limit the generalizability of the study findings. The study also adopted a retrospective design; thus, selection bias is inevitable. We hope to conduct a multi-center, large-sample, prospective study in the future to obtain sufficient objective evidence.

In conclusion, for patients with esophageal cancer and malignant airway stenosis, TAIC is safe and effective; thus, it is worthy of clinical application.

## Data availability statement

The original contributions presented in the study are included in the article/supplementary material. Further inquiries can be directed to the corresponding author.

## Ethics statement

The studies involving human participants were reviewed and approved by Ethics Committee of Zhengzhou University (Ethical approval code: SS-2018-22). The patients/participants provided their written informed consent to participate in this study.

## Author contributions

GZ: Conceptualization, methodology, writing—original draft. MY: Conceptualization, methodology. WH: Methodology. CL: Methodology, project administration. ZL: Investigation, visualization. YM: Formal analysis, investigation, visualization. XL: Writing—reviewing and editing. SW: Software. GW: Funding acquisition, supervision, writing—reviewing and editing. All authors contributed to the article and approved the submitted version.
